# Changes in vigilance, self rated sleep and state anxiety in military personnel in India following yoga

**DOI:** 10.1186/s13104-018-3624-y

**Published:** 2018-07-28

**Authors:** Shirley Telles, Ram Kumar Gupta, Sadhna Verma, Niranjan Kala, Acharya Balkrishna

**Affiliations:** Patanjali Research Foundation, Patanjali Yogpeeth, Haridwar, Uttarakhand 249405 India

**Keywords:** Vigilance, Insomnia, Yoga, Military personnel

## Abstract

**Objectives:**

To study the effects of 9 days of yoga on self-rated sleep, state anxiety and performance in a vigilance test among border security force (BSF) personnel. Seven hundred and twenty-two BSF personnel took part in the trial. They were all males, with an average age of 30.9 ± 7.4 years. All of them were involved in guarding the country’s border. They were deputed for 9 days residential training in yoga. Before and after training they were assessed for self-rated sleep, state anxiety and vigilance.

**Results:**

The results suggest the benefits of yoga in BSF personnel. The BSF personnel showed a significant increase in scores in the vigilance test, a decrease in state anxiety, and improved self-rated sleep.

## Introduction

Military personnel face different levels of stress based on the nature of the role assigned to them [1]. The factors which could influence stress levels are the work load, the exposure to direct combat and the place to which the personnel are deployed [2]. In India the border security force (BSF) are a central armed force whose responsibility includes guarding India’s territorial borders and preventing conflict with other nations [3]. The BSF are most often stationed at high altitudes (i.e., 9000 ft above sea level), where they face social isolation while needing to be hyper vigilant [4]. Being hyper vigilant for long periods can negatively impact physical and mental health with impaired cognition (i.e., attention, concentration and memory) and sleep disorders [5]. The most common sleep disorder is insomnia. Apart from this, vigilance or heightened awareness [6] is associated with increased sympathetic nervous system activity [7], which over a period of time could lead to hypertension and other disorders [8].

Yoga has been used to help military personnel adapt to high altitude [9]. The benefits included quicker adaptation to high altitude, lower sympathetic activity, improved performance at a submaximal level of exercise, better joint flexibility, as well as reduced anxiety and depression with better mental functioning.

Yoga practice helped to perform well in a vigilance task [10] while decreasing sympathetic activity [11]. Apart from increasing vigilance without the associated negative effects, yoga practice positively impacted the quality of sleep in people with normal sleep [12] and in those with chronic insomnia [13]. Yoga practice was shown to improve sleep efficiency and reduce sleep onset latency among other benefits.

BSF personnel have been shown to have a high incidence of anxiety [4]. Yoga practice was associated with reduced state and trait anxiety when practiced for an hour daily for 10 days [14]. This was also seen when military personnel stationed in an army base in north India, who were naïve to yoga, showed reduced state anxiety and better performance in an attention task after a 2 h yoga session [15].

Hence in separate studies yoga practice has been shown to (i) improve vigilance while reducing sympathetic activity, (ii) reduce anxiety and (iii) improve the quality of sleep.

Considering that the BSF personnel are in a state of hypervigilance, with insomnia and anxiety, the present study was designed to assess whether they would benefit from a 9 day residential yoga program with respect to (i) performance in a vigilance task, (ii) state anxiety and (iii) sleep.

## Main text

### Methods

#### Study design and recruitment of research participants

Seven hundred and twenty-two BSF personnel with ages 20–56 years (group mean age ± SD; 30.9 ± 7.4 years) completed the assessments. They were receiving training in yoga at a residential centre in north India. To be included in the trial participants had to (i) be male, (ii) have at least 8 h of duty in a day and (iii) complete the questionnaires correctly. The participants had an experience of 9.2 ± 7.2 years being in the BSF. The majority of participants were non gazetted officers (626 were constables and the rest were head constables) with a minimum of 10–12 years of education. They were all fluent in Hindi, but able to understand and read elementary English. Participation in the study was entirely voluntary. The entire group agreed to participate. The participants’ signed informed consent was explained in Hindi and English and the study was approved by the institutions ethics committee (PRF/16/0014).

The study was a single group longitudinal study. All participants had been deputed to learn yoga hence there was no comparison group to receive an alternate intervention.

#### Assessments

On the day preceding the first day of training and on the day after the ninth day of training participants were assessed for (i) vigilance (ii) state anxiety and (iii) self rated sleep.

(i) Vigilance was assessed using a standard digit vigilance test (DVT) which consists of an A4 size sheet with 1500 digits randomly arranged from 1 to 9 on it in 30 columns and 50 rows [16]. This test measures sustained attention and psychomotor speed [17]. The participants were instructed in Hindi to cancel the target digits ‘6’ and ‘9’ wherever they appeared in the test. Missing any target digits and cancellation of other digits were counted as errors. The score obtained was the total number of digits cancelled minus the errors. The time given to complete the task was 8 min. Participants were familiar with the numbers written as Arabic numerals.
(ii) State anxiety was assessed using the standardized Spielberger’s state trait anxiety inventory (STAI-S) [18] which is a 4-point Likert scale and comprises 20 items to describe the feelings at the moment of testing. The test was administered in Hindi using a standardized version [15]. The items of the inventory are: (1) I feel calm, (2) I feel secure, (3) I am tense, (4) I am regretful, (5) I feel at ease, (6) I feel upset, (7) I am presently worrying over possible misfortunes, (8) I feel rested, (9) I feel anxious, (10) I feel comfortable, (11) I feel self confident, (12) I feel nervous, (13) I am jittery, (14) I feel high strung, (15) I am relaxed, (16) I am content, (17) I am worried, (18) I feel over-exited and rattled, (19) I feel joyful and (20) I feel pleasant. The participants had to choose one out of the four options provided for each item i.e., not at all, somewhat, moderately so, and very much so. The scores range from 20 to 80.
(iii) Self-rated sleep quality was assessed using the Sleep Rating Questionnaire (SRQ) which is standardized for use in an Indian population and is in Hindi [19]. The SRQ consists of one open-ended (item 4) and six close-ended items which are: (1) Approximately how long in minutes does it take you to fall asleep? (2) How many minutes do you sleep each night? (3) How many times (if any) do you wake up during the night? (4) What are the usual reasons for waking up if you do so? (5) Do you feel rested in the morning (yes/no)? (6) Do you sleep in the day time (yes/no)? (7) If your answer to question 6 was ‘yes’, for how long does your daytime sleep last in minutes?

#### Intervention

The yoga intervention was for 240 min in each day. The yoga sessions were conducted in a large hall that can accommodate 4000 people. The 722 BSF personnel were seated in 20 rows and 37 columns (i.e., each row having 37 persons) separated by 3.0 ft distance between them. The dimension of the hall was 500 × 350 ft. Each yoga session was led by an expert in yoga who has over 45 years of experience in practicing yoga, having learned yoga in childhood. In addition there were 20 yoga teachers who had experience of 5 years or more of teaching yoga who were assigned to a single row. Audiovisual aids were used to ensure that the yoga expert who was at the front of the hall on an elevated platform, was visible and audible to all participants. Instructions were given in Hindi using a microphone and 24 speakers (placed on either side of the hall), each with an output of 136 dB. Two LED screens (10 × 16 feet each) were placed at the front of the hall, approximately 320 ft away from the last row. The yoga sessions included a universal prayer for 3 min, warming up for 10 min, and loosening exercises for 10 min, yoga postures (*asanas*) for 50 min, voluntarily regulated breathing techniques (*pranayamas*) for 35 min, and guided relaxation for 10 min followed by a concluding prayer, which was also universal. The timings for yoga sessions were between 05:00–07:00 h and 16:00–18:00 h daily for 9 days. The details of the yoga session are given in Table 1.

**Table 1:** Details of a yoga practice session

S. no.		Yoga practice	Duration (min)
1	Starting	Universal prayer	3
2	Warming up	Sun salutation *(Surya namaskara)*	10
3	Loosening exercises	Neck rotation	3
		Knee and ankle rotation	3
		Shoulder rotation	3
		Butterfly pose	1
4	Standing postures	Swaying palm tree pose *(Tirryaktadasana)*	3
		Tree pose *(Vrikshasana)*	3
		Feet and hands posture *(Padahastasana)*	2
5	Sitting postures	Frog posture *(Mandukasana)*	3
		Rabbit posture *(Sasakasana)*	3
		Sitting lateral twisting posture *(Vakrasana)*	5
		Cow face posture *(Gomukhasana)*	5
6	Prone postures	Crocodile posture *(Makarasana)*	5
		Cobra posture *(Bhujangasana)*	3
		Half-locust posture *(Salabhasana)*	3
7	Supine postures	Half-plough posture *(Ardhhalasana)*	3
		Rotating leg pose *(Padavritasana)*	6
		Leg circling pose *(Dwichakrasana)*	6
8	Relaxation	Supine relaxed posture *(Shavasana)* with breath awareness	5
9	Yoga breathing series *(pranayamas)*	Yoga bellows type breathing *(Bhastrika)*	3
		High frequency yoga breathing *(Kapalabhati)*	10
		*Bahyavriti pranayama*	3
		Victorious breath *(Ujjayi)*	3
		Alternative nostril yoga breathing *(Anlom*-*vilom)*	10
		Bee breathing practice *(Bhramari)*	3
		Om chanting *(Udgeeth)*	3
10	Relaxation	Supine relaxed posture *(Shavasana)* with breath awareness	5
11		Concluding prayer	2

Total duration of the session was 120 min and the same practices have been repeated during the evening session too

The participants were provided a shared room for two persons with an attached bathroom within the same campus as the yoga hall. Apart from the yoga practice sessions participants were given lectures (3 h/day) on principles of stress reduction and coping with stress based on principles of yoga (*Patanjali’s Yoga Sutras*; by the sage Patanjali, *Circa* 900 B.C.) [20]. The rest of the day was for them to complete personal chores (e.g., laundry), reading or studying, walking or playing outdoor games. The campus includes a dining hall, cafeteria, grounds to walk in, and a shop where all basic requirements can be bought. Participants were required to stay in the residential campus during the yoga training period.

#### Data analysis

Paired t-tests were performed to compare vigilance scores, state anxiety and self-rated sleep before and after the yoga practice in BSF personnel. Two items of the SRQ (i.e., number 5 and number 6) were measured on a nominal scale (i.e., yes = 1, no = 0) and analyzed with Pearson’s Chi square test.

### Results

The BSF personnel showed a significant increase in the scores of the digit vigilance test (t = 15.42, P < 0.001) and a significant decrease in state anxiety (t = 21.33, P < 0.001) after 9 days of yoga. The total duration of nocturnal sleep increased significantly (t = 11.44, P < 0.001). There was also a significant decrease in the time taken to fall asleep (t = 7.43, P < 0.001), number of arousals during the night (t = 11.18, P < 0.001) and duration of day time sleep (t = 15.66, P < 0.001). The two nominal scores i.e., (i) ‘the feeling of being rested in the morning’ increased (χ^2^ = 115.47, P < 0.001, Cramer’s V = 0.28) and (ii) ‘sleeping in the day’ decreased (χ^2^ = 39.78, P< 0.001, Cramer’s V = 0.17) after yoga compared to before. The details are given in Table 2. One item of the SRQ that is ‘reason for night time awakenings’ was open ended. These reasons were most often (i) using the rest room, (ii) bad dreams, or (iii) thirst. This did not change after yoga.

**Table 2:** Changes in vigilance, state anxiety and self rated sleep quality before and after 9 days of yoga intervention

Variables	Yoga intervention					
	Before	After	t value	χ^2^ value	Cohen’s d/Cramer’s V	P value
Vigilance scores	284.46 ± 45.98	307.15 ± 22.13	15.42	–	0.63^b^	< 0.001
State anxiety scores	38.61 ± 9.68	30.98 ± 9.03	21.33	–	0.82^b^	< 0.001
Time taken to fall asleep (min)	29.69 ± 21.22	20.27 ± 25.25	7.43	–	0.40^b^	< 0.001
Total duration of nocturnal sleep (min)	276.68 ± 63.25	308.20 ± 51.02	11.54	–	0.55^b^	< 0.001
Number of arousals during the night	1.34 ± 1.04	0.88 ± 0.85	11.18	–	0.48^b^	< 0.001
Duration of daytime sleep (min)	72.86 ± 42.25	44.57 ± 35.89	15.66	–	0.72^b^	< 0.001
Feeling of being rested in the morning (number of participants who reported ‘yes’)^a^	476	645	–	115.47	0.28^c^	< 0.001
Sleeping in the day (number of participants who reported ‘yes’)^a^	599	504	–	39.78	0.17^c^	< 0.001

Values are group mean ± SD

^a^The remainder of the participants reported ‘No’

^b^Cohen’s d

^c^Cramer’s V

The effect sizes obtained in the present study for state anxiety and self-rated sleep are higher than those reported in volunteers after 7 days of yoga in identical conditions [12].

### Discussion and conclusion

Seven hundred and twenty-two border security force personnel (BSF) received 9 days of residential training in yoga. At the end of the training the BSF personnel showed an increase (by an average of 31 min) in total amount of nocturnal time spent asleep, decrease in sleep onset latency (by an average of 9 min), reduced day time sleep and nocturnal arousals. Their state anxiety levels were decreased by 20% while their scores in a vigilance test were significantly better.

The BSF require to be alert and vigilant while they guard the country’s borders. Prolonged alertness and attention can influence the parts of the brain which help to stay awake, as well as those involved in actively inducing sleep [17]; in addition, neural pathways which actively promote sleep are inhibited by sustained attention [21]. Alertness and attention are also associated with increased sympathetic activity which would further promote wakefulness and prevent sleep [22]. As yoga practice is known to increase parasympathetic activity [23] this could reduce physiological arousal associated with sympathetic activation (e.g., increased heart rate, cardiac output and blood pressure, among other changes). With a predominance of parasympathetic tone as well as the decrease in state anxiety seen in the BSF personnel, it is possible that in the CNS too there would be changes, with activation of sleep inducing areas. This is a speculation, but it is supported by numerous EEG studies which have shown an increase in slow EEG frequencies [24] as well as increased cortical gamma amino butyric acid measured by NMRS as an immediate effect of yoga practice [25, 26]. These mechanisms could explain the improvement in sleep and decrease in anxiety after 9 days of yoga. In turn, decreased anxiety could improve performance in the vigilance test suggesting better concentration [27].

Hence the results suggest that intensive yoga training can help BSF personnel to perform with better sustained attention and psychomotor speed in a vigilance test, have better sleep and reduced state anxiety. This could help them perform their duties better. The BSF have a job which is of great importance, is very demanding and requires optimal physical and mental fitness [4]. There are comparable jobs in other countries which require physical fitness, courage, emotional stability and the ability to be attentive and act quickly. This could include people in the military, in the police force as well as other jobs such as pilots (both military and commercial). Hence the findings of the present study have a broader relevance.

## Limitations

The limitations include (i) the absence of a control group, (ii) the improved digit vigilance test performance could have been a learning effect and (iii) since the data was not blind scored there could be bias.

Hence though the participants followed a regular yoga schedule and showed significant changes the results cannot be considered conclusive.

## Abbreviations

BSF: border security force
SRQ: Sleep Rating Questionnaire
DVT: digit vigilance test
STAI-S: Spielberger’s state trait anxiety inventory
CNS: central nervous system
EEG: electroencephalogram
NMRS: nuclear magnetic resonance spectroscopy
